# Impact of Transport Media on IL‐33 Levels in Samples From Patients With Viral Respiratory Infections

**DOI:** 10.1155/jimr/3274290

**Published:** 2026-09-15

**Authors:** Daniela Moreno-Tapia, Yaneisi Vázquez, Valentina Pavez, Omar P. Vallejos, José Acevedo-López, Camila Pardo, Felipe A. Cancino, Ana María Contreras, Marcela Ferrés, Dona Benadof, Ana María Guzmán, Francisca Valdivieso, Mauricio Farfán, María Luisa Rioseco, Valentina Gutiérrez, Pablo J. Bertrand, Eduardo Espejo, Angélica Domínguez, Cecilia V. Tapia, Pablo A. González, Hernán F. Peñaloza, Alexis M. Kalergis, Susan M. Bueno

**Affiliations:** ^1^ Millennium Institute on Immunology and Immunotherapy, Facultad de Ciencias Biológicas, Pontificia Universidad Católica de Chile, Santiago, Chile, imii.cl; ^2^ Laboratorio de Infectología y Virología Molecular, Escuela de Medicina, Pontificia Universidad Católica de Chile, Santiago, Chile, uc.cl; ^3^ Departamento de Enfermedades Infecciosas e Inmunología Pediátricas, Escuela de Medicina, Pontificia Universidad Católica de Chile, Santiago, Chile, uc.cl; ^4^ Laboratorio Clínico y Microbiología Hospital Roberto del Río, Santiago, Chile; ^5^ Laboratorio Hospital Clínico UC, Departamento de Laboratorios Clínicos, Facultad de Medicina, Escuela de Medicina, Pontificia Universidad Católica de Chile, Santiago, Chile, uc.cl; ^6^ Laboratorio de Microbiología, Hospital Luis Calvo Mackenna, Santiago, Chile; ^7^ Facultad de Medicina, Universidad San Sebastián, Sede Patagonia, Puerto Montt, Región de Los Lagos, Chile, uss.cl; ^8^ Unidad de Infectología Pediátrica, Hospital Dr. Sótero del Río, Santiago, Chile; ^9^ División de Pediatría, Facultad de Medicina, Departamento de Enfermedades Respiratorias Pediátricas, Pontificia Universidad Católica de Chile, Santiago, Chile, uc.cl; ^10^ Laboratorio Clínico, Hospital Dr. Sótero del Río, Santiago, Chile; ^11^ School of Public Health, Facultad de Medicina, Pontificia Universidad Católica de Chile, Santiago, Chile, uc.cl; ^12^ Laboratorio Clínico Hospital de Carabineros, Santiago, Chile; ^13^ Departamento de Laboratorios Clínicos, Escuela de Medicina, Facultad de Medicina, Pontificia Universidad Católica de Chile, Santiago, Chile, uc.cl; ^14^ Departamento de Endocrinología, Facultad de Medicina, Escuela de Medicina, Pontificia Universidad Católica de Chile, Santiago, Chile, uc.cl

**Keywords:** IL-33 detection, NPS, PBS, respiratory tract infection, UTM

## Abstract

Interleukin‐33 (IL‐33) is an IL‐1 family cytokine that activates various immune cells and induces both systemic and localized Th2 responses during infection. An association between IL‐33 and its suppresion of tumorigenicity 2 (ST2) receptor with inflammation during respiratory tract infection has been previously reported. Furthermore, some data suggest that during respiratory infections, IL‐33 may serve as an indicator of disease severity, as high levels of this cytokine have been found in nasopharyngeal swab (NPS) samples of infants with lower respiratory tract disease caused by respiratory syncytial virus (RSV). As viral diagnosis is mostly compartmentalized in NPS, in this report, we aimed at detecting IL‐33 in NPS from patients with respiratory tract infections that were collected in two different transport media (universal transport medium [UTM], and phosphate‐buffered saline [PBS]). IL‐33 levels were evaluated in 230 NPS samples, 121 obtained in PBS, and 109 in UTM. The samples were tested for RSV, metapneumovirus, adenovirus (ADV), influenza, and parainfluenza. In this cohort, higher levels of IL‐33 were detected in virus‐positive samples collected in PBS compared to virus‐negative samples collected in the same transport medium. In addition, RSV‐ and FLU‐positive samples collected in PBS showed higher measured IL‐33 levels than those collected in UTM. No significant differences were found for the other viruses included in this study. These findings highlight the importance of considering sample collection and transport conditions when interpreting IL‐33 measurements in NPS, particularly in future studies evaluating IL‐33 as a potential severity‐associated biomarker during RSV or influenza infection.

## 1. Introduction

Interleukin‐33 (IL‐33) is a cytokine belonging to the IL‐1 family, and its receptor, suppression of tumorigenicity 2 (ST2), is part of the IL‐1 receptor family (IL‐1R) [1]. IL‐33 has been described as an alarmin [2], as it is produced by endothelial cells, epithelial cells, and fibroblasts during inflammatory processes and can activate different types of ST2‐expressing immune cells, including group 2 innate lymphoid cells (ILC2s), regulatory T cells (Tregs), mast cells, CD4^+^ Th2 cells, and B cells, among others [3]. IL‐33 and ST2 have been widely studied in inflammatory conditions, including allergies [4–6], asthma [7, 8], fibrotic diseases [9, 10], autoimmune diseases [11], and cardiovascular diseases [12, 13]. Based on these studies, particularly in respiratory allergic diseases, IL‐33 has been proposed as a potential biomarker of disease prognosis [5].

Recent studies have also suggested an association between IL‐33 signaling and respiratory tract inflammation during viral infections [14]. In hospitalized children under 2 years of age with acute bronchiolitis, plasma IL‐33 levels were elevated during rhinovirus infection or rhinovirus‐respiratory syncytial virus (RSV) coinfection [15]. In another study, children with RSV‐induced acute bronchiolitis showed a predominant Th2 response over a Th1 response, together with increased serum levels of IL‐33, IL‐10, and IL‐4 compared with controls [16]. IL‐33 has also been detected in respiratory samples from children with acute respiratory viral infections. For instance, IL‐33 and other cytokines were elevated in nasopharyngeal swabs (NPS) from children aged 3–24 months during acute respiratory virus infections [17]. In addition, IL‐33 levels were significantly increased in respiratory samples, including NPS or nasopharyngeal aspirates, from RSV‐positive patients with lower respiratory tract infections, such as bronchiolitis, compared with patients with upper respiratory tract infections [17–19]. Together, these studies support the relevance of evaluating IL‐33 in respiratory samples during viral infections.

In the case of influenza A virus (FLU), in vitro infection of human lung epithelial cells (A549) increased the IL‐33 expression [20]. In addition, a study conducted in children under 2 years of age hospitalized with bronchiolitis caused by respiratory viruses reported detectable IL‐33 levels in nasopharyngeal aspirates from children infected with RSV, human metapneumovirus (hMPV), parainfluenza virus (PIV), human rhinovirus (HRV), and human bocavirus (HBoV) but not in nasopharyngeal aspirates from healthy infants [21].

The identification of respiratory viruses in respiratory secretions can be achieved using polymerase chain reaction (PCR)‐based molecular methods or antigen detection assays [22]. Different transport media are routinely used for sample collection across health care centers. Among the most commonly used media for NPS samples are universal transport medium (UTM), viral transport medium (VTM), and phosphate‐buffered saline (PBS) [23, 24]. The performance of UTM, PBS, and VTM is comparable in terms of maintaining viral RNA integrity and stability for PCR‐based detection [25]. However, few studies have evaluated whether these transport media similarly preserve other biomolecules of interest, such as proteins and cytokines, whose measurement in respiratory samples may be relevant for future biomarker studies.

In the current study, we evaluated IL‐33 levels detected in NPS samples from patients with respiratory tract infections collected in PBS or UTM at different health care centers in Santiago, Chile.

## 2. Materials and Methods

### 2.1. Ethical Approval

The protocols associated with obtaining and maintaining nasopharyngeal samples at the Molecular Pathogenesis Laboratory of the School of Biological Sciences, Pontificia Universidad Católica de Chile, previously collected in three health centers in Santiago, were reviewed and approved by the Institutional Scientific Ethics Committee of Health Sciences of the Pontificia Universidad Católica de Chile (Numbers 15‐336 and 191210009), approved on December 17, 2015, and June 7, 2016, respectively, and by the Scientific Ethics Committee of the South East Metropolitan Health Service (Number 2016001), approved on August 25, 2016. The approved protocols included a waiver of informed consent.

### 2.2. Characteristics of Samples

Two hundred thirty (230) nasopharyngeal secretion samples were collected through NPS from different patients attending three health care centers in Santiago, Chile. Samples were collected and analyzed by direct immunofluorescence (DIF) or PCR to diagnose viral infection. The remaining part of the sample was delivered at 2–8°C to the Molecular Pathogenesis Laboratory of the School of Biological Sciences at Pontificia Universidad Católica de Chile. Samples were delivered anonymously, so the clinical data of each patient were unknown; only viral diagnosis and age were available. Negative and positive samples for RSV, hMPV, adenovirus (ADV), FLU (A and B), and PIV were collected in 2019 at the following clinical centers: the Clinical Hospital Red Salud UC‐CHRISTUS (*n* = 109), the Roberto del Río Hospital (RRH; *n* = 57), and the Sótero del Río Hospital (SRH; *n* = 64). Demographic information of the subjects, viral diagnosis, and collection media are described in Table 1.

**Table 1:** Demographic information, viral diagnosis by viruses, and collection media. Chile, between 2019 and 2021.

Viral diagnosis	Age at viral diagnosis	NPS obtained in PBS	NPS obtained in UTM
		(*n* = 121)	(*n* = 109)
RSV	*n* total	**20**	**24**
	≤5 years	18	21
	6–64 years	2	1
	≥65 years	0	2
hMPV	*n* total	**21**	**17**
	≤5 years	16	12
	6–64 years	2	4
	≥65 years	3	1
ADV	*n* total	**20**	**12**
	≤5 years	17	11
	6–64 years	3	1
	≥65 years	0	0
FLU	*n* total	**20**	**20**
	≤5 years	10	2
	6–64 years	9	15
	≥65 years	1	3
PIV	*n* total	**20**	**16**
	≤5 years	16	11
	6–64 years	1	5
	≥65 years	3	0
Negative	*n* total	**20**	**20**
	≤5 years	15	3
	6–64 years	5	9
	≥65 years	0	8

*Note:* The values in bold represent the total *n* for each viral diagnosis.

Abbreviations: ADV, adenovirus; FLU, influenza virus; hMPV, human metapneumovirus; NPS, nasopharyngeal swabs; PBS, phosphate‐buffered saline; PIV, parainfluenza virus; RSV, respiratory syncytial virus; UTM, universal transport medium.

### 2.3. Clinical Sample Collection and Processing

**Figure 1:**
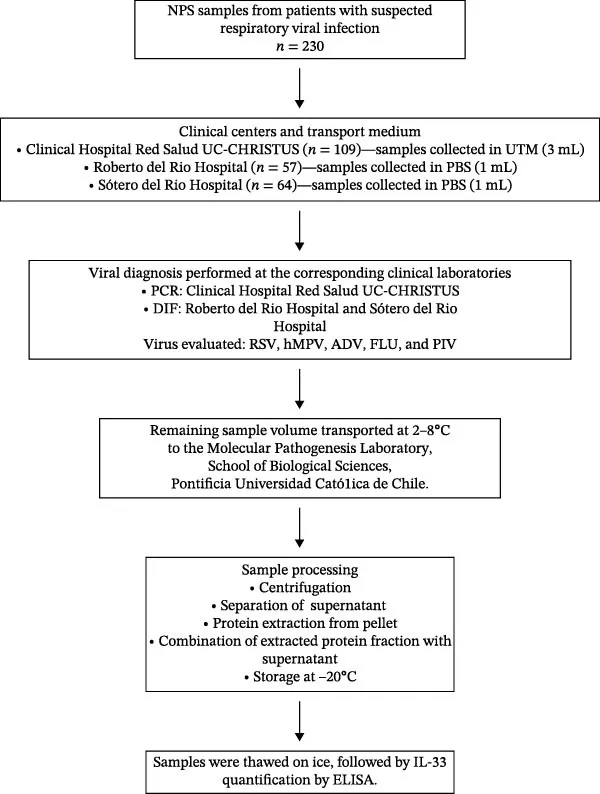
Workflow of NPS sample collection, viral diagnosis, processing, and IL‐33 measurement. A total of 230 NPS samples were collected from patients with suspected respiratory viral infection attending three health care centers in Santiago, Chile. Samples from Clinical Hospital Red Salud UC‐CHRISTUS were collected in UTM and analyzed for viral diagnosis by PCR, whereas samples from Roberto del Río Hospital and Sótero del Río Hospital were collected in sterile PBS and analyzed by DIF. The remaining sample volume was transported at 2–8°C to the Molecular Pathogenesis Laboratory, processed for protein extraction, stored at −20°C, thawed on ice, and analyzed for IL‐33 levels by ELISA.

NPS were collected using sterile swabs that were inserted into both nostrils until reaching the posterior nasopharyngeal area. The samples obtained in UTM (BD UVT standard kit, BD Universal Viral Transport system; BD, USA; SKU 220,221) (*n* = 109) were collected in 3 mL of media, following standard clinical procedures, and subsequently analyzed in the laboratory of Infectiology and Molecular Virology and the Clinical Laboratory of the Clinical Hospital Red Salud UC‐CHRISTUS for viral diagnosis. The samples obtained in sterile PBS (*n* = 121) were collected in 1 mL, following standard clinical procedures, and analyzed in the Clinical Laboratory of RRH and the Clinical Laboratory of SRH for viral diagnosis. Upon receipt of the sample, the remaining volume was transferred to a 1.5 mL tube and centrifuged at 4000 g for 4 min at room temperature. The supernatant from each sample was separated and stored at 4°C for no longer than 24 h before protein extraction. Protein extraction was performed according to the protocol described by Gonzalez et al. [26]. Briefly, the pellets were treated with 100 µL of RIPA Buffer (Pierce, Thermo Scientific #89900, USA) containing protease inhibitor cocktail tablets (Roche #11836145001, USA) for 15 min at 4°C, with vortexing every 5 min. Then, the samples were centrifuged at 4000 g for 4 min, and the supernatant was collected. The supernatant obtained after protein extraction was combined with the supernatant obtained after the first centrifugation and stored at −20°C until the IL‐33 measurement. PBS‐ and UTM‐collected samples were stored at −20°C for comparable periods before the analysis. Samples were thawed directly for IL‐33 quantification by ELISA and were not subjected to repeated freeze–thaw cycles before the IL‐33 measurement. The overall workflow for sample collection, transport, viral diagnosis, processing, and IL‐33 measurement is summarized in Figure 1.

### 2.4. Viral Diagnosis (PCR or DIF)

In the laboratory of Infectious Diseases and Molecular Virology and the Clinical Laboratory of the Clinical Hospital Red Salud UC‐CHRISTUS, each virus was identified using the Luminex RPP Molecular Panel PCR kit or the FilmArray Pneumonia Panel (BioFire Diagnostics), respectively. Respiratory pathogens include influenza A virus (FLU) with H1 and H3 subtypes, influenza B viruses, PIVs 1, 2, 3, and 4, RSV (types A and B), ADVs, and hMPV, among others. In the clinical laboratories of RRH and SRH, the five viruses (FLU type A and B, RSV, PIV, ADV, and hMPV) were detected by DIF.

### 2.5. IL‐33 Measurement

The levels of IL‐33 in the NPS samples were detected using an ELISA kit (Peprotech, #900‐K398) according to the manufacturer’s instructions. The dynamic detection range of the kit was 32 to 4000 pg/mL. IL‐33 levels were determined in positive and negative samples obtained in PBS and UTM for each virus evaluated in this study (RSV, hMPV, ADV, FLU, and PIV), as identified by PCR or DIF.

Due to the limited volume available after NPS sample processing, each clinical sample was analyzed once by ELISA without prior dilution. IL‐33 concentrations were calculated from the standard curve according to the manufacturer’s instructions. Values calculated below or above the dynamic detection range were retained in the analysis because they corresponded to the values obtained from the standard curve under the experimental conditions used. However, these values were considered outside the assay’s validated quantification range and were therefore interpreted with caution. Values reported as 0 corresponded to undetectable or below‐range signals and were not interpreted as an exact biological absence of IL‐33.

Because NPS samples collected in PBS and UTM were eluted in different final volumes (1 mL for PBS and 3 mL for UTM), IL‐33 concentrations measured in UTM‐collected samples were adjusted by applying a dimensionless dilution factor of 3. This normalization accounts for the threefold larger collection volume used for UTM samples and expresses IL‐33 values as concentrations equivalent to those that would have been obtained if the samples had been collected in 1 mL.

### 2.6. Statistical Methodology

IL‐33 values were summarized as medians and interquartile ranges and further analyzed by transport medium, viral diagnosis, and virus‐specific status. In all comparisons, virus‐negative samples were defined as samples negative for all viruses evaluated in this study, whereas virus‐positive samples were defined as samples positive for at least one of the viruses of interest.

Nonparametric tests were used to compare IL‐33 values between groups. For comparisons between two independent groups, two‐sided Mann–Whitney tests were performed. For comparisons involving more than two groups, the Kruskal–Wallis test followed by Dunn’s multiple‐comparisons post hoc test was used. IL‐33 levels were compared between PBS‐ and UTM‐collected samples, between virus‐positive and virus‐negative samples within each transport medium, and between PBS‐ and UTM‐collected virus‐positive samples for each virus evaluated.

**Figure 2:**
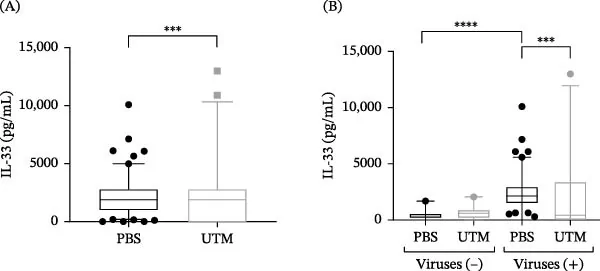
IL‐33 levels in NPS samples collected in PBS or UTM. IL‐33 (pg/mL) levels measured by ELISA in NPS samples collected in PBS or UTM that tested positive or negative for RSV, hMPV, ADV, FLU, or PIV (*n* = 230). (A) IL‐33 levels (pg/mL) of total NPS samples (positive and negative for viruses) collected in PBS (*n* = 121) or UTM (*n* = 109). (B) Comparison of IL‐33 levels (pg/mL) between the samples collected in PBS negative for all viruses (*n* = 20) and positive (*n* = 101) for RSV, hMPV, ADV, FLU, or PIV, and those collected in UTM negative for all viruses (*n* = 20) and positive (*n* = 89) for viruses. Each data point represents an individual clinical NPS sample analyzed once for IL‐33. Exact *p*‐values, effect sizes, and 95% confidence intervals for the main comparisons are provided in Table S1. Data are presented as the median and 5^th^–95^th^ percentile values, shown as horizontal and square bars, respectively. Statistical analysis was performed using nonparametric tests: a two‐sided Mann–Whitney test for (A) and the Kruskal–Wallis test followed by Dunn’s multiple‐comparisons post hoc test for (B) ( ^∗∗∗∗^ *p*  < 0.0001,  ^∗∗∗^ *p*  < 0.001).

**Figure 3:**
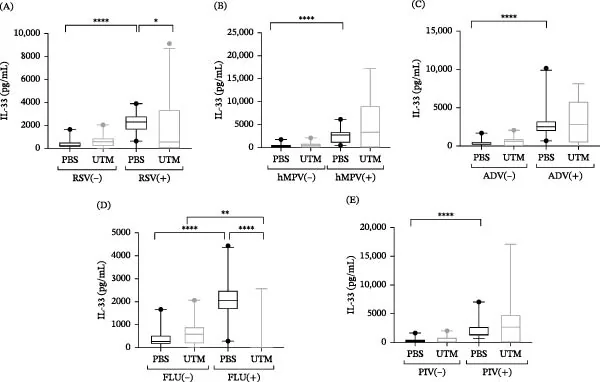
IL‐33 levels in NPS samples collected in PBS or UTM according to viral diagnosis. IL‐33 levels (pg/mL) in NPS samples assessed by ELISA in (A) RSV(+) in PBS (*n* = 20) or UTM (*n* = 24). (B) hMPV(+) in PBS (*n* = 21) or UTM (*n* = 17). (C) ADV(+) in PBS (*n* = 20) or UTM (*n* = 12). (D) FLU(+) in PBS (*n* = 20) or UTM (*n* = 20). (E) PIV(+) in PBS (*n* = 20) or UTM (*n* = 16). Negative samples correspond to IL‐33 levels (pg/mL) evaluated in a pool of negative samples for all viruses (RSV[−], hMPV[−], ADV[−], FLU[−], and PIV[−]) in PBS or UTM (*n* = 20 for each medium). Each data point represents an individual clinical NPS sample analyzed once for IL‐33. Exact *p*‐values, effect sizes, and 95% confidence intervals for the main comparisons are provided in Table S1. The data shown correspond to median and 5^th^–95^th^ percentile values, represented by horizontal and square bars, respectively. Statistical analysis was performed using the nonparametric Kruskal–Wallis test followed by Dunn’s multiple‐comparisons post hoc test ( ^∗∗∗∗^ *p*  < 0.0001,  ^∗∗∗^ *p*  < 0.001,  ^∗∗^ *p*  < 0.01,  ^∗^ *p*  < 0.05).

**Figure 4:**
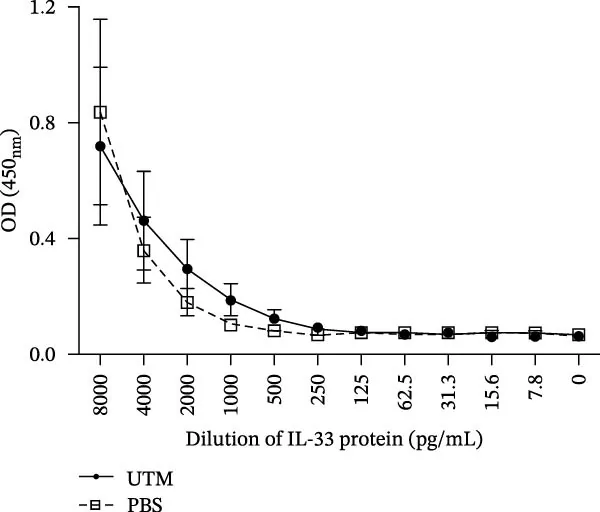
Evaluation of recombinant IL‐33 protein diluted in PBS and UTM. A commercial ELISA was performed with a Peprotech kit. The plate was activated with 3 mg/mL of capture antibody. Nonspecific sites were blocked with a blocking solution (10% fetal bovine serum diluted in 1× phosphate‐buffered saline). Then, IL‐33 protein was incubated at 8000–7.8 pg/mL diluted in UTM media or PBS. A blank well (without protein) was incubated with a detection antibody at a concentration of 0.25 μg/mL as a negative control. The HRP‐conjugated detection antibody was incubated at a dilution of 1:2000. The data shown on the *Y*‐axis correspond to the optical density (OD) measured at 450 nm. Data shown are means ± SEM of two independent experiments. Statistical analysis was performed using two‐way ANOVA followed by Sidak’s multiple‐comparisons test, with transport medium and recombinant IL‐33 concentration as factors.

Effect sizes for the main comparisons shown in Figures 2 and 3 were reported as Hodges–Lehmann median differences with 95% confidence intervals and are provided in Table S1. For the recombinant IL‐33 assay shown in Figure 4, OD_450_ values were compared using two‐way ANOVA, followed by Sidak’s multiple‐comparisons test, with transport medium and recombinant IL‐33 concentration as factors. Data from this assay are presented as means ± SEM from two independent experiments.

## 3. Results

### 3.1. Comparison of IL‐33 Detection Between Clinical Samples Collected in UTM or PBS

NPS samples from 230 subjects were recruited between 2019 and 2021. The subjects’ demographic information, sample sizes for each virus, and collection media are detailed in Table 1.

When comparing the transport media for NPS sample collection, the levels of IL‐33 detected were significantly higher in samples from subjects collected in PBS (*n* = 121) compared to those samples from subjects collected in UTM (*n* = 109), regardless of the diagnosis, whether the result is positive or negative for all viruses (Figure 2A). Because PBS‐collected samples were obtained from two clinical centers, SRH and RRH, we also compared IL‐33 levels between PBS‐SRH and PBS‐RRH samples. No statistically significant differences were observed between these groups, suggesting comparable IL‐33 detection among PBS‐collected samples from both centers (Figure S1).

Next, we compared IL‐33 levels detected by ELISA in virus‐positive and virus‐negative samples. As shown in Figure 2B, samples positive for any virus (*n* = 101) collected in PBS exhibited significantly higher IL‐33 levels compared to virus‐negative samples (*n* = 20). When samples were collected in UTM, virus‐positive samples (*n* = 89) showed a nonstatistically significant trend toward higher detected IL‐33 levels compared with virus‐negative samples (*n* = 20) (Figure 2B). When comparing virus‐positive NPS samples collected in PBS (*n* = 101) with those collected in UTM (*n* = 89), significantly higher IL‐33 levels were observed in PBS‐collected samples (Figure 2B). Effect size estimates for the main comparisons shown in Figure 2 are provided in Table S1.

### 3.2. Virus‐Specific Levels of IL‐33 in NPS of Virus‐Infected Patients

Next, we analyzed the levels of IL‐33 in negative and positive samples for RSV, hMPV, ADV, PIV, and FLU (Figure 3A–E).

We observed that the level of this cytokine was significantly higher in samples positive for each of the five viruses when collected in PBS than in negative samples collected in the same medium. This significant difference was not observed when comparing negative and positive samples collected in UTM, except for FLU‐positive samples, which unexpectedly showed lower detected levels of IL‐33 than FLU‐negative samples (Figure 3D). When comparing median IL‐33 levels between NPS samples collected in PBS and UTM for each virus, RSV‐positive samples showed that PBS‐collected samples were 4 times higher than UTM‐collected samples. A similar situation was observed for FLU, where the median IL‐33 values in samples collected in PBS were up to twice those in samples collected in UTM (Table 2). No differences were observed in the median values of samples positive for hMPV, ADV, and PIV (Table 2) when comparing those collected in PBS versus UTM. Effect size estimates for the virus‐specific comparisons shown in Figure 3 are provided in Table S1.

**Table 2:** Description of IL‐33 levels obtained from NPS and transported in PBS or UTM medium by viral diagnosis.

Diagnosis	PBS (IL‐33 pg/mL)			UTM (IL‐33 pg/mL)		
	Median	P25	P75	Median	P25	P75
Positive for RSV						
Yes	2317.2	1644.6	2808.3	589.5	0.000	3320.2
No	270.6	161.8	529.8	599.1	185.4	891.0
* p*‐Value	0.0000002	—	—	0.736	—	—
Positive for hMPV						
Yes	2674.3	1010.6	3356.6	3309.0	109.5	9001.5
No	270.6	161.8	529.8	599.1	185.4	891.0
* p*‐Value	0.0000008	—	—	0.050	—	—
Positive for ADV						
Yes	2531.5	1922.6	3191.8	2784.1	425.2	5776.5
No	270.6	161.8	529.8	599.1	185.4	891.0
* p*‐Value	0.0000002	—	—	0.017	—	—
Positive for FLU (A or B)						
Yes	2064.0	1683.3	2480.2	0.000	0.000	0.000
No	270.6	161.8	529.8	599.1	185.4	891.0
*p*‐Value	0.000001	—	—	0.001	—	—
Positive for PIV						
Yes	1403.7	1083.7	2778.0	2581.5	0.00000	4719.0
No	270.6	161.8	529.8	599.1	185.4	891.0
*p*‐Value	0.000001	—	—	0.261	—	—

*Note:* P25, 25th percentile; P75, 75th percentile.

Abbreviations: ADV, adenovirus; FLU, influenza virus; hMPV, human metapneumovirus; IL‐33, interleukin‐33; NPS, nasopharyngeal swabs; PBS, phosphate‐buffered saline; PIV, parainfluenza virus; RSV, respiratory syncytial virus; UTM, universal transport medium.

Next, we evaluated the median detection rate based on significant differences in IL‐33 detection between virus‐positive and virus‐negative samples collected in PBS. We determined that hMPV induced the highest levels of IL‐33 in NPS samples among all viruses evaluated in the study (Table 3). A ranking of IL‐33 production, ordered from highest to lowest, in virus‐positive versus virus‐negative samples collected in PBS is shown in Table 3. The ranking results for the samples collected in UTM are not shown because no significant differences were found between virus‐positive and virus‐negative subjects (Figure 3).

**Table 3:** The ratio between the median levels detected in positive and negative samples for each virus in PBS‐transported samples.

Virus	Median IL‐33 values	Median IL‐33 value	Ratio	Ranking
	(Virus‐positive)	(Virus‐negative)^a^		
hMPV	2674.3	270.6	9.88	1
ADV	2532		9.35	2
RSV	2317.5		8.56	3
FLU	2064		7.62	4
PIV	1404		5.18	5

Abbreviations: ADV, adenovirus; FLU, influenza virus; hMPV, human metapneumovirus; IL‐33, interleukin 33; PBS, phosphate‐buffered saline; PIV, parainfluenza virus; RSV, respiratory syncytial virus.

^a^Value obtained from 20 samples collected in PBS.

### 3.3. Detection of Recombinant IL‐33 Levels in Two Buffer Types (UTM or PBS)

To determine whether the collection buffer interferes with IL‐33 detection by ELISA, 12 concentrations of recombinant IL‐33 protein (ranging from 8000 to 7.8 pg/mL) were evaluated after dilution in PBS or UTM medium. The results show that the detection of the recombinant protein is very similar regardless of the dilution medium (PBS or UTM) (Figure 4). However, some differences are observed at concentrations between 4000 and 500 pg/mL, with OD values for UTM higher than those for PBS. On the other hand, this trend changes at a concentration of 8000 pg/mL, where the detection with PBS is greater than with UTM.

## 4. Discussion

This study aimed to evaluate how sample collection may influence IL‐33 detection in NPS samples from patients with respiratory viral infections. IL‐33 participates in inflammatory processes, including asthma [7, 8], and has been associated with disease severity in some respiratory viral infections, such as RSV [17–19]. Therefore, it is critical to analyze the preanalytical conditions that may affect IL‐33 measurement in NPS samples from patients with viral respiratory tract infections. Defining these conditions is important for future studies evaluating this cytokine in the context of respiratory viral infections and may contribute to the development of standardized tests aimed at providing clinically relevant information on disease severity.

Our results showed a higher detection of IL‐33 in NPS samples collected in PBS (regardless of their viral diagnosis) than in UTM samples. This significant difference is maintained when comparing NPS samples collected in PBS from subjects with a viral infection (virus‐positive) to those collected with UTM who also presented a viral infection. Also, significantly higher levels of IL‐33 were obtained in RSV‐ and FLU‐positive samples when collected in PBS compared to UTM. Therefore, sample collection and transport conditions should be considered when measuring cytokines such as IL‐33 in studies evaluating severity‐associated biomarkers during RSV or FLU infections. However, given that the transport medium was associated with clinical center and laboratory workflow, the observed differences cannot be attributed exclusively to the composition of PBS or UTM. Rather, the differences observed may reflect the combined influence of the transport medium, clinical matrix composition, laboratory workflow, and other preanalytical factors. To further evaluate whether center‐related handling and processing could influence IL‐33 detection among samples collected in the same transport medium, we compared PBS‐collected NPS samples from SRH and RRH. No statistically significant differences in IL‐33 levels were observed between PBS‐SRH and PBS‐RRH samples (Figure S1), suggesting that, when the same transport medium was used, center‐specific sample handling and processing did not appear to be a major source of variability in IL‐33 detection.

Among samples collected in PBS, virus‐positive NPS samples showed consistently higher detected IL‐33 levels than virus‐negative samples for the respiratory viruses included in this study (RSV, hMPV, ADV, FLU, and PIV). These findings are consistent with previous studies reporting increased IL‐33 levels in respiratory samples from infants with severe viral bronchiolitis caused by RSV and rhinovirus compared with subjects with a negative viral diagnosis [21]. Similarly, elevated IL‐33 levels have been described in nasopharyngeal samples from infants under 2 years of age with RSV‐induced bronchiolitis compared with RSV‐negative samples [17]. Additional studies have also reported an association between IL‐33 detection and increased risk of mechanical ventilation in hospitalized children with acute lower respiratory tract infections caused by RSV, HRV, ADV, or FLU [18], increased IL‐33 expression in the lungs of FLU‐infected mice [20], and IL‐33 induction in hMPV‐infected human alveolar epithelial cells [27]. Regarding the elevated levels of IL‐33 detected in FLU‐positive samples collected in PBS, these findings may be influenced, at least in part, by the heterogeneous ages of the patients evaluated, as previous studies have reported that the immune response varies considerably with the stage of immunological development [28].

Therefore, IL‐33 induction has already been described in several respiratory viral infections. The main contribution of the present study is not to establish IL‐33 elevation as a novel finding across respiratory viruses but rather to highlight that IL‐33 detection in NPS samples may vary according to sample collection, transport medium, and other preanalytical conditions. Further studies using standardized collection and processing protocols are needed to determine how transport conditions influence IL‐33 measurement and to evaluate its potential relationship with clinical outcomes.

Although our data show that lower levels of IL‐33 were detected in clinical samples collected in UTM compared to PBS, when recombinant IL‐33 protein was diluted in UTM or PBS, no differences in IL‐33 detection were observed between media in the concentration range of 0–250 pg/mL. However, at concentrations above 500 pg/mL, IL‐33 detection, measured as optical density at 450 nm (OD_450_), was slightly higher when recombinant IL‐33 was diluted in UTM than in PBS.

While this recombinant IL‐33 assay in PBS and UTM allowed us to evaluate whether the transport media alone affected IL‐33 detection by ELISA, this approach does not fully reproduce the complexity of clinical NPS samples. According to the manufacturer’s information, the UTM used in this study contains protein for stabilization, antibiotics to minimize bacterial and fungal contamination, and a buffer to maintain a neutral pH. Although these components are intended to preserve respiratory pathogens during transport, their interaction with the clinical nasopharyngeal matrix cannot be excluded. The nasopharyngeal matrix contains water, mucins, globular proteins, salts, DNA, lipids, cells, and cellular debris, which may influence cytokine recovery or detection [29]. In this context, UTM components may interact with mucus proteins, cellular material, or soluble inflammatory mediators, potentially affecting IL‐33 recovery, stability, epitope accessibility, or ELISA detection. Therefore, the differences observed in clinical samples should be interpreted as potentially resulting from the combined influence of transport medium composition, clinical matrix characteristics, laboratory workflow, diagnostic approach, and other preanalytical factors, rather than from a direct effect of the transport medium alone.

Finally, UTM media have been reported to interfere with certain diagnostic tests as they may lead to false‐positive results in viral diagnoses using rapid antigen test devices [30]. It is worth noting that these devices are based on specific monoclonal antibodies. Therefore, it remains possible that transport media, together with the clinical matrix, may influence cytokine detection in sandwich ELISA assays; however, this possibility requires further validation in clinical NPS extracts.

## 5. Limitations of the Study

When interpreting these findings, several limitations must be considered. First, the small sample sizes of positive and negative samples for the different viruses (hMPV, PIV, RSV, FLU, and ADV) may reduce the robustness of our analyses. Second, the choice of transport medium (PBS vs. UTM) depended on the clinical collection site, laboratory workflow, and viral diagnostic method (PCR panel vs. DIF), thereby excluding intracenter comparisons. In addition, differences in diagnostic sensitivity between PCR‐based detection and DIF may have influenced the classification of virus‐negative samples, and potential false‐negative cases cannot be completely excluded. In addition, this study did not include samples from a single patient collected in both sampling media simultaneously, which would have allowed paired comparisons between PBS and UTM. Third, baseline age imbalances were present between the cohorts, particularly among the FLU and negative control groups, which could influence baseline cytokine levels.

Fourth, due to the limited volume available from processed NPS samples, IL‐33 levels were measured without prior sample dilution. As a result, some calculated values fell outside the validated dynamic range of the ELISA kit, including values below the range reported as 0. These values were retained to preserve the complete dataset but should be interpreted with caution, as the statistical analyses compare the distribution of calculated IL‐33 values under the experimental conditions used rather than providing precise quantification of values outside the validated assay range. Therefore, potential matrix interferences or volume‐dilution effects cannot be entirely discarded. In addition, spike‐and‐recovery and linearity‐of‐dilution experiments using clinical NPS extracts collected in PBS and UTM were not performed. Thus, the extent to which the clinical nasopharyngeal matrix may affect IL‐33 recovery, assay linearity, or ELISA detection in each transport medium could not be directly determined. Although the recombinant IL‐33 assay in PBS and UTM allowed us to evaluate the effect of the transport media under controlled conditions, it does not fully reproduce the complexity of clinical NPS samples.

Fifth, because the clinical samples were fully anonymized, detailed information on the origin of the sample, such as gender, patient status (hospitalized or ambulatory), days of hospitalization, and other clinical severity data, was unavailable. Thus, a correlation between the observed variations in IL‐33 levels and disease severity was not possible. Finally, coinfections with other viruses or pathogens were not evaluated in the present analysis. Therefore, we could not determine whether some virus‐specific groups included patients with additional respiratory pathogens, nor could we perform mono‐infection analyses. Because coinfections may modify local inflammatory responses and cytokine levels in respiratory samples, their potential influence on IL‐33 detection cannot be excluded.

## 6. Conclusions

Using biomarkers such as IL‐33, which has been associated with disease severity in respiratory viral infections, could be a valuable tool to improve patient management during epidemic or seasonal outbreaks. However, further studies are needed to evaluate how sample collection, transport conditions, and processing workflows are associated with IL‐33 measurement in respiratory clinical samples. Studies should be performed to determine whether this cytokine correlates with clinical severity parameters when samples are obtained in either PBS or UTM. In this study, significantly higher levels of IL‐33 were detected in RSV‐ and FLU‐positive samples collected in PBS than in UTM. No significant differences were found for the other viruses included in this study. Therefore, sample collection, transport medium, processing workflow, and other preanalytical conditions should be carefully considered when interpreting cytokine measurements such as IL‐33 in NPS samples from patients with RSV or FLU infection.

## Author Contributions

Daniela Moreno‐Tapia, Yaneisi Vázquez, Valentina Pavez, Omar P. Vallejos, Camila Pardo, and Susan M. Bueno conceived and planned the experiments. Daniela Moreno‐Tapia, Yaneisi Vázquez, Valentina Pavez, Camila Pardo, and Omar P. Vallejos carried out the experiments. Ana María Contreras, Marcela Ferrés, Dona Benadof, Ana María Guzmán, Francisca Valdivieso, Valentina Gutiérrez, and Eduardo Espejo contributed to sample collection and preparation. Daniela Moreno‐Tapia, Yaneisi Vázquez, Valentina Pavez, Omar P. Vallejos, José Acevedo‐López, Felipe A. Cancino, Alexis M. Kalergis, Pablo A. González, Hernán F. Peñaloza, Camila Pardo, and Susan M. Bueno contributed to the interpretation of the results. Daniela Moreno‐Tapia, Yaneisi Vázquez, Valentina Pavez, Omar P. Vallejos, José Acevedo‐López, Camila Pardo, and Susan M. Bueno took the lead in writing the manuscript. José Acevedo‐López, Felipe A. Cancino reanalyzed the data provided in the revised version of the manuscript. All authors provided critical feedback and contributed to shaping the research, data analysis, and final manuscript.

## Funding

This study was supported by Grants 11IDL2‐10668 and 15VEIID‐45704 from the INNOVA‐CORFO Program of the Chilean Ministry of Economy, Grants 13CTI‐21526‐P5 and 23CTEC‐250091‐P2 from CORFO Program of Chilean Ministry of Economy, and by the ANID‐ICM Millennium Institute on Immunology and Immunotherapy (Grant ICN2021_045). Grant TA25I10042 from the ANID IDeA I+D Tecnologías Avanzadas Program (Concurso IDeA I+D Tecnologías Avanzadas 2025).

## Disclosure

Patent applications have been filed by Pontificia Universidad Católica de Chile to protect the monoclonal antibodies and the immunochromatographic device. Felipe A. Cancino is an ANID PhD fellow #21251401.

## Ethics Statement

The protocols associated with this study were reviewed and approved by the Institutional Scientific Ethics Committee of Health Sciences of the Pontificia Universidad Católica de Chile (Numbers 15‐336 and 191210009), and for the Scientific Ethics Committee of the South East Metropolitan Health Service (Number 2016001). All procedures were conducted in accordance with the current Tripartite Guidelines for Good Clinical Practices and the Declaration of Helsinki.

## Consent

The approved protocols included a waiver of informed consent because the study used anonymized data and/or samples, ensuring that no personally identifiable information from participants was collected or disclosed.

## Conflicts of Interest

The authors declare no conflicts of interest.

## Supporting Information

Additional supporting information can be found online in the Supporting Information section.

## Supporting information


**Supporting Information** The Supporting Information accompanying this article contains one figure and one table. Figure S1 shows the comparison of IL‐33 levels measured by ELISA in PBS‐collected NPS samples from two clinical centers, Sótero del Río Hospital (PBS‐SRH, *n* = 64) and Roberto del Río Hospital (PBS‐RRH, *n* = 57), evaluating whether center‐specific sample handling influenced IL‐33 detection when the same transport medium was used. Table S1 provides the effect size estimates for the main IL‐33 comparisons presented in Figures 2, 3, and S1, reported as Hodges–Lehmann median differences with their corresponding 95% confidence intervals and adjusted *p*‐values.

## Data Availability

The raw data supporting the conclusions of this article will be made available by the authors, without undue reservation.
