# Supplementary material for: Impact of Transport Media on IL‐33 Levels in Samples From Patients With Viral Respiratory Infections
Source: J Immunol Res. 2026 Sep 15;2026:3274290. doi: 10.1155/jimr/3274290 (PMC13579107; doi:10.1155/jimr/3274290)
Supplement: Supplementary file 1 [file JIMR-2026-3274290-s001.docx]

**Supplementary Material**

**Supplementary Figure S1.** IL-33 levels in PBS-collected NPS samples from two clinical centers. IL-33 (pg/mL) levels measured by ELISA in NPS samples collected in PBS from Hospital Sótero del Río (PBS-SRH, n = 64) or Hospital Roberto del Río (PBS-RDRH, n = 57) (n = 121). Each data point represents an individual clinical NPS sample analyzed once for IL-33. Exact p-value, effect size, and 95% confidence interval for the comparison are provided in Supplementary Table 1. Data are presented as the median and 5th–95th percentile values, shown as horizontal and square bars, respectively. Statistical analysis was performed using a two-sided Mann–Whitney test. ns, not significant.

**Supplementary Table 1**. Effect size estimates for the main IL-33 comparisons shown in Figures 2, 3 and S1.

| **Figure** | **Comparison** | **Median group 1** | **Median group 2** | **Hodges–Lehmann median difference** | **95% CI** | **p-value / adjusted p-value** |
| --- | --- | --- | --- | --- | --- | --- |
| Figure 2A | PBS vs UTM, all samples | 1887 | 574.5 | 638.0 | 276.0 to 1069 | 0.0006 |
| Figure 2B | PBS virus-negative vs UTM virus-negative | 270.7 | 599.1 | -300.9 | -563.3 to 26.25 | >0.9999 |
| Figure 2B | PBS virus-positive vs UTM virus-positive | 2137 | 414.0 | 959.3 | 379.0 to 1439 | 0.0005 |
| Figure 2B | PBS virus-positive vs PBS virus-negative | 2137 | 270.7 | 1784 | 1385 to 2251 | <0.0001 |
| Figure 2B | UTM virus-positive vs UTM virus-negative | 414.0 | 599.1 | 0.000 | -312.0 to 1989 | 0.3114 |
| Figure 3A | PBS RSV(+) vs PBS RSV(-) | 2318 | 270.7 | 1839 | 1444 to 2433 | <0.0001 |
| Figure 3A | UTM RSV(+) vs UTM RSV(-) | 589.5 | 599.1 | 0.000 | -549.8 to 2240 | >0.9999 |
| Figure 3A | PBS RSV(+) vs UTM RSV(+) | 2318 | 589.5 | 1300 | -570.0 to 1887 | 0.0117 |
| Figure 3A | PBS RSV(-) vs UTM RSV(-) | 270.7 | 599.1 | -300.9 | -563.3 to 26.25 | >0.9999 |
| Figure 3B | PBS hMPV(+) vs PBS hMPV(-) | 2674 | 270.7 | 2287 | 1069 to 2863 | <0.0001 |
| Figure 3B | UTM hMPV(+) vs UTM hMPV(-) | 3309 | 599.1 | 2710 | 0.000 to 7500 | 0.1135 |
| Figure 3B | PBS hMPV(+) vs UTM hMPV(+) | 2674 | 3309 | -1060 | -5385 to 1033 | >0.9999 |
| Figure 3B | PBS hMPV(-) vs UTM hMPV(-) | 270.7 | 599.1 | -300.9 | -563.3 to 26.25 | >0.9999 |
| Figure 3C | PBS ADV(+) vs PBS ADV(-) | 2532 | 270.7 | 2093 | 1693 to 2661 | <0.0001 |
| Figure 3C | UTM ADV(+) vs UTM ADV(-) | 2784 | 599.1 | 1919 | 246.0 to 2814 | 0.0504 |
| Figure 3C | PBS ADV(+) vs UTM ADV(+) | 2532 | 2784 | 191.5 | -1241 to 1689 | >0.9999 |
| Figure 3C | PBS ADV(-) vs UTM ADV(-) | 270.7 | 599.1 | -300.9 | -563.3 to 26.25 | >0.9999 |
| Figure 3D | PBS FLU(+) vs PBS FLU(-) | 2064 | 270.7 | 1722 | 1394 to 2041 | <0.0001 |
| Figure 3D | UTM FLU(+) vs UTM FLU(-) | 0.000 | 599.1 | -563.3 | -844.5 to -143.3 | 0.0066 |
| Figure 3D | PBS FLU(+) vs UTM FLU(+) | 2064 | 0.000 | 1912 | 1649 to 2321 | <0.0001 |
| Figure 3D | PBS FLU(-) vs UTM FLU(-) | 270.7 | 599.1 | -300.9 | -563.3 to 26.25 | >0.9999 |
| Figure 3E | PBS PIV(+) vs PBS PIV(-) | 1404 | 270.7 | 1128 | 847.0 to 1732 | <0.0001 |
| Figure 3E | UTM PIV(+) vs UTM PIV(-) | 2582 | 599.1 | 1655 | -312.0 to 2729 | 0.8877 |
| Figure 3E | PBS PIV(+) vs UTM PIV(+) | 1404 | 2582 | 242.6 | -1746 to 1176 | 0.5777 |
| Figure 3E | PBS PIV(-) vs UTM PIV(-) | 270.7 | 599.1 | -300.9 | -563.3 to 26.25 | >0.9999 |
| Figure S1 | PBS-SRH vs PBS RDRH | 1903 | 1767 | 399.0 | -77.0 to 885.0 | 0.0959 |

Effect sizes are reported as Hodges–Lehmann median differences with 95% confidence intervals. For Figure 2A and Supplementary Figure S1, p-values correspond to the two-sided Mann–Whitney test. For Figures 2B and 3A–E, adjusted p-values correspond to Dunn’s multiple-comparisons post-hoc test following Kruskal–Wallis analysis.
